# Effect of Hyperbaric Oxygenation on Blood Cytokines and Arginine Derivatives; No Evidence for Induction of Inflammation or Endothelial Injury

**DOI:** 10.3390/jcm10235488

**Published:** 2021-11-23

**Authors:** Jacek Siewiera, Michał Smoleński, Natalia Jermakow, Jacek Kot, Klaudia Brodaczewska, Jacek Turyn, Magdalena A. Zabielska-Kaczorowska, Nils Ludwig, Mirosław J. Szczepański

**Affiliations:** 1 Department of Hyperbaric Medicine, Military Institute of Medicine, 04-349 Warsaw, Poland; jsiewiera@wim.mil.pl (J.S.); njermakow@gmail.com (N.J.); 2 Department of Biochemistry, Medical University of Warsaw, 02-097 Warsaw, Poland; mszczepanski@wum.edu.pl; 3 National Centre for Hyperbaric Medicine, Institute of Maritime and Tropical Medicine, Medical University of Gdansk, 81-519 Gdynia, Poland; jkot@gumed.edu.pl; 4 The Laboratory of Molecular Oncology and Innovative Therapies, Military Institute of Medicine, 04-349 Warsaw, Poland; kbrodaczewska@gmail.com; 5 Department of Biochemistry, Medical University of Gdansk, 80-211 Gdansk, Poland; jacek.turyn@gumed.edu.pl (J.T.); magdalena.zabielska@gumed.edu.pl (M.A.Z.-K.); 6 Department of Physiology, Medical University of Gdansk, 80-211 Gdansk, Poland; 7 Department of Oral and Maxillofacial Surgery, University Hospital Regensburg, 93053 Regensburg, Germany; Nils.Ludwig@klinik.uni-regensburg.de

**Keywords:** ADMA, SDMA, cytokines, hyperbaric oxygenation, HBOT

## Abstract

(1) Background: Hyperbaric oxygen therapy (HBOT) uses 100% oxygen delivered at 1.5–3 times the atmospheric pressure in a specialised chamber to achieve supraphysiological oxygen tension in blood and tissues. Besides its target, HBOT may affect inflammation, endothelial function or angiogenesis. This study analysed the effect of HBOT on blood concentrations of factors that may affect these processes in patients with necrotizing soft-tissue infections (NSTI), aseptic bone necrosis (ABN) and idiopathic sudden sensory neural hearing loss (ISSNHL). (2) Methods: Concentrations asymmetric dimethylarginine (ADMA) and other arginine derivatives were measured with liquid chromatography/mass spectrometry, whereas ELISA was used to quantitate vascular endothelial growth factor (VEGF) and cytokines (IL-1, IL-4, IL-6, IL-10, TGF-β) before and after HBOT in 80 patients (NSTI *n* = 21, ISSNHL *n* = 53, ABN *n* = 6). (3) Results: While some differences were noted between patient groups in ADMA and other arginine derivatives as well as in cytokine concentrations, HBOT did not affect any of these parameters. (4) Conclusions: While cytokines and arginine derivatives concentrations were modified by underlying pathology, hyperbaric oxygenation did not immediately modify it suggesting that it is neutral for inflammation and is not inducing endothelial injury.

## 1. Introduction

Hyperbaric oxygen therapy (HBOT) uses 100% oxygen that is delivered at 1.5–3 times the atmospheric pressure in a specialised chamber. This procedure has been proven effective in decompression sickness, carbon monoxide poisoning and sudden sensorineural hearing loss [1]. It is also considered as an adjunct to surgical and pharmacological interventions such as problematic wounds [2]. At normal atmospheric pressure, the oxygen in human body is provided to organs and tissues with haemoglobin in red blood cells distributed through the circulatory system. Prolonged inflammation induces oxygen deficiency in the affected tissue due to destruction of capillaries, oedema, bacteria, and production of free radicals in the hypoxic conditions [3]. HBOT significantly increases the concentration of oxygen in blood plasma which diffuses to the damaged tissue from the capillaries [4], ultimately inducing a variety of biochemical changes including the promotion of angiogenesis, prevention of the release of free radicals and proteases, elimination of anaerobic bacteria, and increased neutrophil-mediated inflammatory response [5]. Although the aforementioned effects are still only supported by limited data.

The process of angiogenesis is complex with a multitude of factors contributing to its promotion or inhibition. Vascular endothelial growth factor (VEGF) is considered as one of the most important factors and has been the focus of numerous studies [6,7,8,9,10,11]. The initial release of VEGF is caused by the tissue damage and hypoxic environments, which induce migration and proliferation of endothelial cells and formation of new blood vessels. This process involves different cell populations, including lymphocytes, fibroblasts, and macrophages [12]. VEGF expression is affected by several factors such as asymmetrical dimethylarginine (ADMA) and its enantiomer, symmetric dimethylarginine (SDMA), which are products of proteolysis of post-translational methylated proteins. They are also endothelial toxins that block nitric oxide synthase (NOS) and affect production of nitric oxide (NO) from L-Arginine [13]. ADMA and SDMA are metabolised by dimethylarginine dimethyl aminohydrolase (DDAH), which is impaired by oxidative stress [14]. Therefore, it constitutes a useful marker for verifying the hypoxic and inflammatory tissue environment and endothelial functionality. These conditions are influenced by the local oxygen levels and the presence of cytokines such as IL-1, IL-4, IL-6, IL-10, and TGF-β [15]. Although the biochemical pathways are not yet fully understood and require further research, there is some evidence that HBOT modify the pro-inflammatory cytokine synthesis in macrophages [16,17]. It is also known that a prolonged exposure to hyperoxic conditions can potentially have side effects on the human body [18]. It is thus critical to pursue further studies to optimize use of HBOT and to find best approach that will balance safety and effectiveness. The aim of the study was to characterize changes in the concentration of arginine derivatives, VEGF and other cytokines in the blood serum of patients subjected to hyperbaric oxygenation to identify potential effect of HBOT on angiogenesis, inflammation and endothelial injury.

## 2. Materials and Methods

### 2.1. Patients

**Figure 1:**
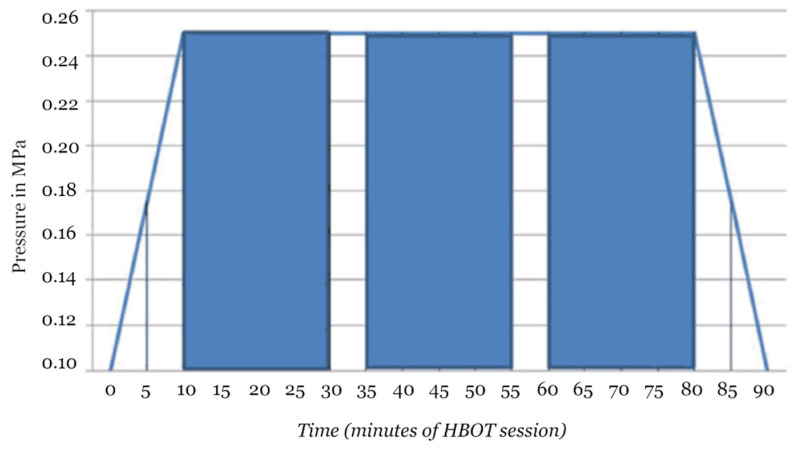
Hyperbaric oxygenation session profile consisting of compression phase, 100% oxygen phase (indicated by blue bars) with two 5 min air breaks and decompression.

Having obtained the approval of the Bioethical Commission of the Military Institute of Medicine (28; 19 June 2019 to J.S.), a blood serum testing of 83 patients of the Department of Hyperbaric Medicine of the Military Institute of Medicine (Warsaw, Poland), treated with hyperbaric oxygenation was performed between June 2019 and January 2020. Treatments were carried out in accordance with the attached hyperbaric oxygenation profile in the number of sessions related to weeks of daily treatment without breaks in the weekend, depending on the type of indication for treatment (Figure 1).

Inclusion criteria were the age between 18 and 85 years old and qualification for hyperbaric oxygenation treatment according to recommendations of European Consensus Conference on Hyperbaric Medicine [1]. Exclusion criteria were: pregnancy, un-controlled asthma, chemotherapy (in particular bleomycin and doxorubicin), chest surgery in the last 6 months, emphysema and bronchiectasis, closed gas spaces within the sinuses of the skull and other regions, heart failure (NYHA III/IV), acute coronary syndrome and fresh focal lesions of the central nervous system. Completion of all treatment sessions was required for inclusion in the study. Patients who did not complete treatment according to the planned protocol, those who formed small groups of patients with a given diagnosis (e.g., radiation cystitis *n* = 2), or were heterogeneous groups of patients with known multimorbidity (e.g., diabetic foot syndrome) were excluded from the statistical analysis.

The analysis involved results of patients with necrotizing soft tissue infection (NSTI) (*n* = 18), idiopathic sudden sensory neural hearing loss (ISSNHL) (*n* = 45), and aseptic bone necrosis (ABN) (*n* = 6). All HBOT sessions took place in a multiplace hyperbaric chamber under the direct supervision of medical staff. Patients breathed 100% O_2_ at 2.5-fold higher pressure than at sea level for 60 min. Each session lasted approximately 90 min with compression, two 5 min air-breaks and decompression. Additional therapy, including local surgical treatment and antibiotic therapy in NSTI and steroid therapy in ISSNHL were administered according to the orders of the attending physicians at the referring wards. NSTI had broad-spectrum antibiotic therapy, followed by targeted antibiotic therapy when microbiological results were available. ISNNHL had systemic steroid therapy administered together with HBOT. No drugs affecting the immune system were used in ABN. Blood was collected before commencing HBOT and after two weeks of therapy, under aseptic conditions, from peripheral veins, in a separate puncture, then centrifuged and frozen until tested at −80 °C. Exact time of collection in relation to HBOT and duration of sample preparation procedure was similar for all collected samples.

Concentrations of arginine derivatives were measured with the use of mass spectrometry (Arginine, Homoarginine, SDMA, ADMA, L-NMMA), whereas concentrations of cytokines (IL-1, IL-10, IL-4, IL-6, TGF-β) and VEGF were measured by means of ELISA before HBOT and two weeks following the first measurement (after 15 HBOT sessions).

A total of 69 patients aged 19–84 years (Median = 49; IQR = 37–62) were covered by the analysis and they had between 15 and 60 (Median = 15; IQR = 15–30; min = 15; max = 60) hyperbaric chamber compressions depending on the type of condition (Table 1).

### 2.2. Enzyme-Linked Immunoabsorbent Assay

For each blood collection, 3 mL of peripheral blood was collected by venepuncture into serum tube (Vacutainer SST™ II Advance, BD, Warsaw, Poland) and incubated at room temperature (RT) for 30 min to allow clot formation. To obtain serum, blood samples were fractionated by centrifugation at 2500× *g* for 15 min at RT. Serum supernatant was aliquoted, frozen and stored at −80 °C. Secreted factors were determined with the use of ELISA method: IL-1beta, VEGF (DuoSet ELISA, R&D, Warsaw, Poland) and IL-6, IL-10, TGF-β as described by manufacturer (eBioscience, Warsaw, Poland). Briefly, serum samples were thawed on ice, centrifuged at 2500× *g* for 15 min at 4 °C, and 100 uL of sample was tested in duplicates on primary antibody coated and blocked 96 well plates. For TGF-β measurement, the sample was initially activated with HCl and then neutralised with NaOH, according to the supplier’s guidelines (eBioscence, Warsaw, Poland). Detection was performed with the use of HRP substrate, TMB, and optical density was detected at plate spectrophotometric reader (Multiskan™ GO, ThermoFisher Scientific, Warsaw, Poland) at 450 nm. For each factor, standard curve using recombinant protein was performed, however, blanked absorbance measurements were used for analysis purposes.

### 2.3. Mass Spectrometry

The concentrations of arginine metabolites were determined using liquid chromatography/ mass spectrometry (LC/MS) as we have described previously [19]. Firstly, an aliquot of serum (25 μL) was spiked with internal standards and deproteinized with 0.1 mL of acetonitrile. The tubes were then centrifuged at 4 °C, 12,000× *g* for 5 min. The supernatant was collected and freeze-dried. Samples were then dissolved in 5 μL of water and analysed with ion-pair LC/MS using 2.5 μm Synergy Hydro-RP 50 × 2.0 mm column (Shimpol, Warsaw, Poland). The mass detector (TSQ Vantage, Thermo, Waltham, MA, USA) with heated electrospray (HESI-2) ion source was operating in positive MS2 mode. The electrospray cone voltage was set at 4.5 kV and a heated capillary temperature was 275 °C. Sheath gas flow was set for 35 arbitrary units. Post column make-up flow of methanol with 0.05% formic acid at 0.2 mL/min was used to improve ionization efficiency. The identity of arginine and its derivatives was confirmed by the similarity of chromatographic retention times, molecular weights, and fragmentation pattern.

### 2.4. Statistical Analysis

The compliance of the sample distributions with the normal distribution was checked with the Shapiro-Wilk test. Due to non-normal distribution, the analysis was performed using non-parametric tests. A non-parametric equivalent analysis of variance (Kruskal–Wallis) was performed to test the differences between the groups of diagnoses in arginine derivative concentrations before and within two weeks since HBOT commencement. The Wilcoxon test was used to check which groups had significant differences before and after HBOT. Spearman’s rank correlation was used to assess the correlation of changes in the concentrations of the tested parameters. Due to sample volume limitations it was not possible to obtain complete analyte pattern in all samples. Actual number of measurements is presented in the Results section. Statistical analyses were performed using R software package, version 4.0.3, R Foundation for Statistical Computing, Austria)’ and RStudio software (version 1.4.1103). We also used libraries such as ggplot and stats to perform plots and statistics.

## 3. Results

### 3.1. Quantification of Arginine Derivatives in the Serum of Patients Treated with HBOT

Statistically significant differences were observed in the concentrations of arginine derivatives between the groups of diagnoses before and after the 15 sessions of HBOT (Table 2).

In pairwise comparisons, statistically significant differences were found with the use of Mann-Whitney U test:

- homoarginine concentrations before HBOT between ABN and ISSNHL (*p* = 0.027, Cohen’s d = 0.067) and between NSTI and ISSNHL (*p* = 0.025, Cohen’s d = 0.064) and after 15 HBOT sessions between NSTI and ISSNHL (*p* = 0.012, Cohen’s d = 0.072);
- L-NMMA concentrations between ISSNHL and NSTI after 15 HBOT sessions (*p* = 0.002, Cohen’s d = 2.323);
- ADMA concentrations between ISSNHL and ABN after 15 HBOT sessions (*p* = 0.036, Cohen’s d = 0.385).

**Figure 2:**
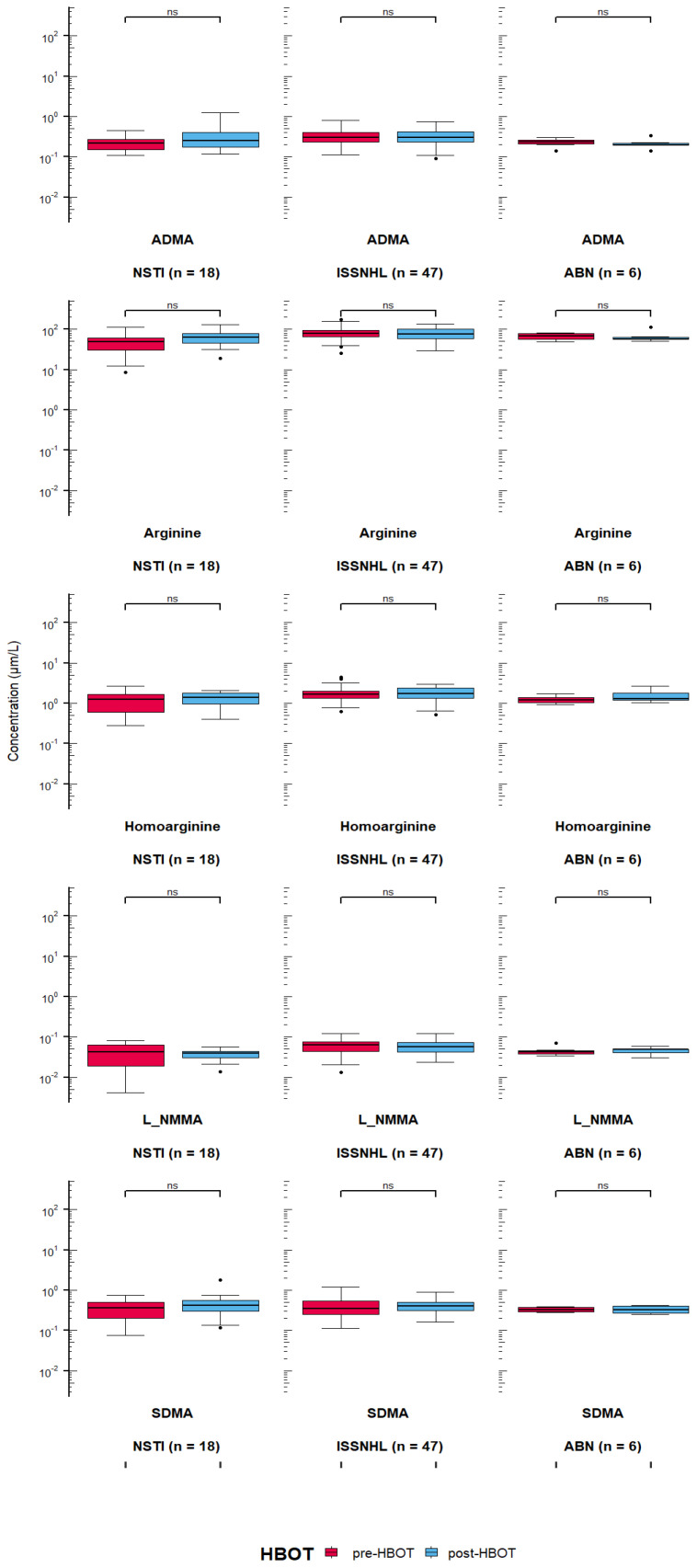
Absence of changes in arginine derivatives when concentrations before hyperbaric oxygen therapy (HBOT) and after 15 HBOT sessions were compared in three patients groups (ns = not significant, ABN—Aseptic bone necrosis, NSTI—Necrotizing soft tissue infection, ISSNHL—Idiopathic sudden sensori-neural hearing loss). Details of statistical analysis are presented in Results section.

The results showed no statistically significant change in mean concentrations of ADMA, SDMA or other arginine derivatives before starting HBOT and after two-weeks of HBOT treatment (with 15 HBOT sessions; Figure 2).

### 3.2. Quantification of Cytokines in the Serum of Patients Treated with HBOT

Patients with both arginine, cytokine and VEGF levels measured were included in the analysis. Cytokine and VEGF levels were measured in 15 NSTI patients (IL-4 and IL-10 in 13 patients), 29 ISSNHL patients (IL-10 in 27 patients) and in 6 ABN patients (IL-10 in 5 patients). Statistically significant differences were observed in the levels of cytokines and VEGF between the groups of diagnoses before and after the 15 sessions of HBOT in IL-6 (Table 3).

In pairwise comparisons (Mann-Whitney U test), statistically significant differences were found in:

- IL-10 level after 15 HBOT sessions between NSTI and ISSNHL (*p* = 0.005, Cohen’s d = 0.0859), and between NSTI and ABN (*p* = 0.01, Cohen’s d = 0.102);
- IL-6 level before HBOT between ISSNHL and ABN (*p* = 0.012, Cohen’s d = 0.209), NSTI and ABN (*p* = 0.0002, Cohen’s d = 0.293), and between ISSNHL and NSTI before (*p* = 0.0002, Cohen’s d = 0.207) and after 15 HBO sessions (*p* = 0.0007, Cohen’s d = 0.1705);
- TGF-β level between ISSNHL and NSTI before HBOT (*p* = 0.005, Cohen’s d = 0.131) and after 15 HBO sessions (*p* = 0.004, Cohen’s d = 0.128), between NSTI and ABN before HBOT (*p* = 0.002, Cohen’s d = 0.108) and after 15 HBO sessions (*p* = 0.0003, Cohen’s d = 0.193), and between ISSNHL and ABN after 15 HBO sessions (*p* = 0.03, Cohen’s d = 0.123);
- VEGF level between ISSNHL and NSTI before HBOT (*p* = 0.002, Cohen’s d = 0.516) and after 15 HBO sessions (*p* = 0.001, Cohen’s d = 0.683), and between NSTI and ABN before HBOT (*p* = 0.0001, Cohen’s d = 0.632) and after 15 HBO sessions (*p* = 0.0009, Cohen’s d = 1.375).

**Figure 3:**
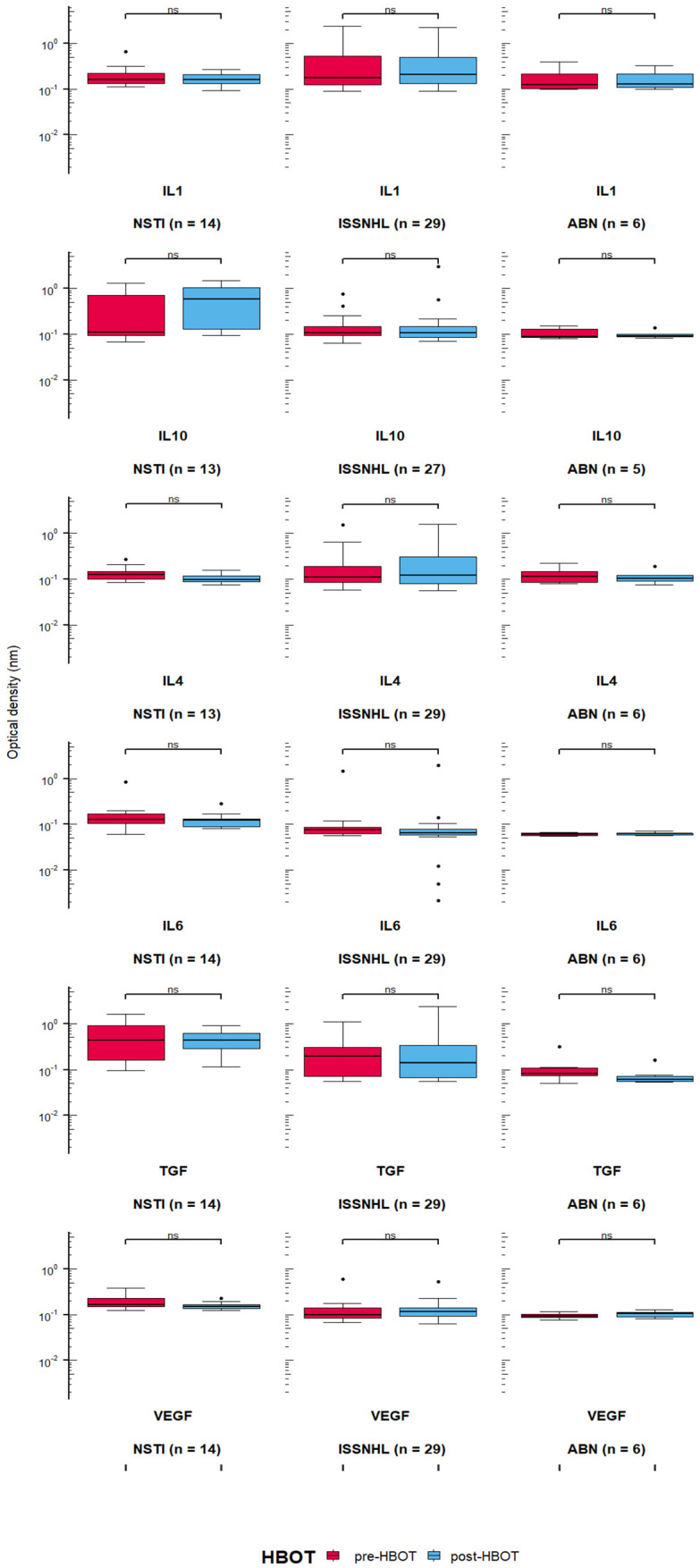
Absence of changes in cytokines levels when concentrations before hyperbaric oxygen therapy (HBOT) and after 15 HBOT sessions were compared in three patients groups (ns = not signifi-cant, ABN—Aseptic bone necrosis, NSTI—Necrotizing soft tissue infection, ISSNHL—Idiopathic sudden sensori-neural hearing loss). Details of statistical analysis are presented in Results section.

The results showed no statistically significant change in IL-1, IL-4, IL-6, IL-10, TGF-β and VEGF in comparisons between levels before starting HBOT and after two-weeks of HBOT treatment (with 15 HBOT sessions; Figure 3). Some differences were borderline in NSTI group, i.e., in IL-4 (*p* = 0.058) and VEGF (*p* = 0.062).

Due to the small number of patients and the lack of compliance with the normal distribution, bootstrapping was performed to test the null hypothesis that there were no differences in the levels of arginine derivatives and cytokines by comparing pre-HBOT and post-HBOT. The analysis was performed with the use of the MKinfer library and the difference of means was compared with the Student’s *t*-test. The results showed that when the sample was multiplied to 1000, significant differences were shown mainly in the NSTI group (Table 4).

### 3.3. Correlations between Levels of Arginine Derivatives and Cytokines in Patients Treated with HBOT

Spearman’s rank correlation analysis was performed to analyse co-variance, i.e., to check whether an increase in concentration involves a decrease or increase in the level of other molecules. The change of the level before HBOT and after 15 HBOT sessions was calculated as a fractional change of post and pre HBOT.

**Figure 4:**
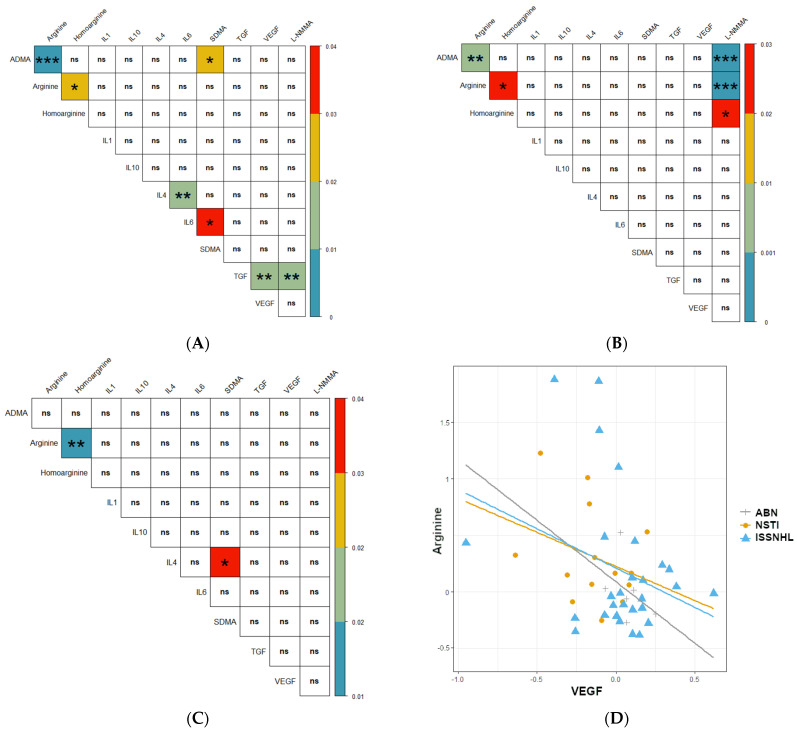
Correlations matrices between levels of arginine derivatives and cytokines in patients treated with HBOT. (**A**) Correlation matrix for necrotizing soft tissue infections. (**B**) Correlation matrix for idiopathic sudden hearing loss. (**C**) Correlation matrix for sterile bone necrosis. (**D**) Co-variability of Arginine and VEGF in the patient groups under study (Necrotizing soft tissue infections (NSTI; *n* = 14); Aseptic bone necrosis (avascular necrosis; ABN; *n* = 6); Idiopathic sudden sensory-neural hearing loss; ISSNHL; *n* = 29). (marks: *** *p* < 0.001; ** *p* < 0.01; * *p* < 0.05; ns-*p* > 0.05).

The results showed that in patients with necrotising soft tissue infection, an increase in L-NMMA entailed an increase in arginine (*p* < 0.0001), homoarginine (*p* = 0.027), and ADMA (*p* < 0.0001); there was also a positive correlation between arginine and homoarginine (*p* = 0.029) and arginine and ADMA (*p* = 0.007); the correlation between homoarginine and IL-10 was at the limit of significance (*p* = 0.050); the other correlations did not reach statistical significance (Figure 4A).

The most correlations between variables occurred among patients with sudden idiopathic hearing loss. The analysis showed positive correlations between Arginine and ADMA (*p* < 0.0001), TGFβ and L-NMMA (*p* = 0.009), Arginine and Homoarginine (*p* = 0.028), SDMA and ADMA (*p* = 0.031), and negative correlations between IL-6 and IL-4 (*p* = 0.001), VEGF and TGFβ (*p* = 0.011), SDMA and IL-6 (*p* = 0.03); the remaining correlations did not reach statistical significance (Figure 4B).

For patients with a diagnosis of sterile bone necrosis (avascular necrosis), a statistically significant result was the positive correlation between IL-4 and SDMA, meaning that in the patients studied, an increase in IL-4 entailed an increase in SDMA (*p* = 0.04); arginine and homoarginine were also shown to be co-variant (*p* = 0.009); the other correlations were not statistically significant (Figure 4C).

Among all the correlations between cytokine levels and Arginine derivatives, the most significant is co-variation between VEGF and Arginine, but no correlation was shown in any of the groups under study. The scatter of the results and the fitted curve are shown in Figure 4D. Although there is no statistical significance, features of co-variation can be observed, but due to the large dispersion of values with small group sizes, the results are not statistically significant.

## 4. Discussion

This study identified that hyperbaric therapy, immediately after 15 sessions, exerts minor effects on endothelium, angiogenesis or inflammation. Clear effects induced by underlying pathologies were not modified by hyperbaric therapy at the biochemical parameters level.

Results of our study do not show statistically significant change in the mean concentration of ADMA, SDMA or other arginine derivatives in the groups of patients subjected to hyperbaric oxygenation. Concentrations of ADMA and other methylated arginine derivatives fluctuate depending on their synthesis as by-products of post-translational methylated proteins proteolysis and elimination via the DDAH pathway. Small amounts can be supplied to the body through the diet. Assuming constant synthesis of methylated arginine derivatives, the increase in ADMA and SDMA concentration consequent to hyperbaric therapy could be expected due to inhibitory effect of oxidative stress on the DDAH enzymatic system. However, this effect was not observed in vivo in patients subject to hyperbaric therapy.

ADMA, SDMA, L-NMMA are important contributors to nitric oxide (NO) metabolism and its physiological functions including control of angiogenesis. NO production considered as major aspect of endothelial function, depends on substrate availability: L-Arginine metabolized by nitric oxide synthase (NOS) [20,21,22]. Both ADMA and L-NMMA are NOS inhibitors, and an increase in serum concentrations entails a decreased release of endothelial NO and narrowed vascular lumen. Both ADMA and the SDMA enantiomer are perceived not only as indicators of vascular endothelial dysfunction, but also as reliable prognostic markers of numerous cardiovascular diseases such as atherosclerosis, heart failure, stroke and kidney disease [23]. Concentrations of ADMA, SDMA, L-NMMA in serum are primarily defined by hydrolysis through type I DDAH associated with neuronal NOS and type II DDAH associated with the endothelial NOS pathway.

Methylated arginine derivatives were suggested to control angiogenesis and modulate the VEGF action [24]. This mechanism is of particular interest in the formation of blood vessels in tumours but has also been described in tissue ischaemia [25]. There are studies showing that overexpression of DDAH enhances VEGF expression in human and murine endothelial cell lines and that NO signalling is particularly important for VEGF-mediated chemotaxis and angiogenesis [26]. On the other hand, VEGF is an endothelium-specific peptide that stimulates angiogenesis and increases arginine transport in endothelial cells [27]. The results of our study may support the observation that VEGF enhances eNOS activity via modulation of arginine uptake [9]. In the combined group of all patients, the increase in VEGF concentration during hyperbaric therapy also entailed a decrease in free arginine concentration in serum (see Figure 3) which may be explained by increased cellular uptake of arginine as a substrate for eNOS.

This would be in agreement with the claim that in endothelial cells, VEGF induces a dose-dependent increase in intracellular eNOS and is associated with increased NO production. However, it does not explain what mechanism leads to an increase in VEGF itself and whether the observed correlation between an increased VEGF concentration and a decreased arginine concentration in the study group is a result of the hyperoxic hyperbaric oxygen therapy applied or whether it occurs independently as a mechanism of regulation of endothelial nitric oxide synthase activity [28].

Angiogenesis in patients treated with HBOT could start with a delay which does require further monitoring and observations. Although in the study the mechanism was not clearly established, we can conclude that vascular growth is not initiated through vascular damage due to HBOT. This discovery allows for possibility of hyperbaric oxygen therapy in wider range of diseases. Further studies are required to verify possible side effects of HBOT and whether it can accompany other treatments and its likely interactions.

Cytokine levels that were also measured in this study did not demonstrate any statistically significant changes. As the markers of potential inflammation process occurring in patients their importance of observing beneficial progress of HBOT may prove useful in determining the specific parameters of therapy for each individual. Further research in this field is required to determine what other factors can potentially accelerate or decelerate the healing process as well as their positive and negative influence on the damaged tissue during HBOT.

Several correlations were identified between parameters measured in this study. Most of these correlations were restricted to relations within cytokines or within arginine derivatives that given number of comparisons may be the effect of statistical chance. However, some correlations such as relation of homoarginine to IL-6 or IL-4 are quite interesting and may indicate potential link between arginine metabolism and immune function. However, this requires further studies.

## 5. Limitations

The limitations of the conducted study relate to the heterogeneity of patients studied and the complexity of the therapeutic process, diverse comorbities and not controlled variables such as social characteristics of patients. It is difficult therefore to isolate specific effect of HBOT on changes in metabolite concentrations but we tried to limit the interference of drugs and surgical procedures by collecting the blood just before and after HBOT. Lack of healthy subject group is an important limitation of our study, but HBOT is only used in subjects with specific pathologies [18]. Since ISSNHL seem to involve only local changes—this group could be treated as the reference in our study. The levels of several quantified metabolites and cytokines were lower than in ABN and significantly lower in NSTI as expected for these more severe and systemic pathologies. Our major aim was to test changes induced by HBOT in specific groups of patients and not to study general effects of HBOT. Another potential limitation is that the increase in concentrations is relatively small and with relatively small samples this might remain undetected. Our results should be therefore interpreted with caution and with assumption that inflammation or endothelial dysfunction are not prominent effect of HBOT, but minor effects should still be assessed in much larger populations.

## 6. Conclusions

Arginine derivatives concentrations differ between NSTI, ISSNHL, and ABN groups treated with the hyperbaric oxygenation, but the effect of hyperbaric oxygenation was negligible. With the emergence of arginine derivatives as potential markers for vascular damage it is possible to conclude that HBOT as applied in this study does not induce endothelial injury immediately after treatment, although long term effects require further research. The observed co-variation of arginine and VEGF concentrations should contribute to further studies on the clinical relevance of the molecular mechanisms.

## Figures and Tables

**Table 1:** Number of patients and mean age, and number of compressions, divided into the individual diagnoses and sex.

		*n*	Median Age (IQR)	Median HBO Sessions (Min–Max)
Aseptic bone necrosis (ABN)	Males	3	39 (28–54)	60 (60)
	Females	3	57 (52–58)	60 (60)
	All	6	52 (39–57)	60 (60)
Necrotizing soft tissue infection (NSTI)	Males	9	54 (19–70)	30 (15–30)
	Females	9	58 (49–84)	30 (30–60)
	All	18	56 (49–70)	30 (15–60)
Idiopathic sudden sensori-neural hearing loss (ISSNHL)	Males	30	54 (37–69)	15 (8–30)
	Females	15	41 (40–47)	15 (15)
	All	45	47 (37–62)	15 (15–30)

**Table 2:** Statistically significant differences from Kruskall–Wallis test for arginine derivatives.

		H	df	*p*	Cohen’s D
Arginine	pre-HBOT	8.999	2	0.011	0.0015
Homoarginine	pre-HBOT	8.154	2	0.017	0.066
	post-HBOT	6.196	2	0.045	0.0732
L-NMMA	pre-HBOT	7.085	2	0. 029	1.95
	post-HBOT	14.005	2	0.0009	2.4

**Table 3:** Statistically significant differences in cytokine concentrations according to Kruskall–Wallis chi-squared test.

		H	df	*p*	Cohen’s D
IL-6	*pre-HBOT*	31.731	2	<0.00001	0.220
	*post-HBOT*	22.959	2	0.00001	0.182
TGF-β	*pre-HBOT*	10.94	2	0.004	0.137
	*post-HBOT*	15.65	2	0.0004	0.134
VEGF	*pre-HBOT*	10.507	2	0.005	0.540
	*post-HBOT*	10.033	2	0.007	0.716
IL-10	*post-HBOT*	13.945	2	0.0009	0.0904

**Table 4:** Bootstrapping statistics with significant results.

	Group	Estimate	SE	CI Upper	CI Lower	*p*
Arginine	NSTI	−13.087	4.888	−4.142	−22.252	0.002
VEGF	NSTI	0.042	0.019	0.0815	0.010	0.008
ADMA	NSTI	−0.102	0.058	−0.018	−0.233	0.018
Homoarginine	ABN	−0.332	0.154	−0.090	−0.641	0.02
IL4	NSTI	0.031	0.015	0.064	0.006	0.022
SDMA	NSTI	−0.122	0.076	−0.018	−0.292	0.026

## Data Availability

Full dataset of this study is available upon justified request.
